# Development of a Novel Low-Cost Knee Brace to Quantify Human Knee Function During Dynamic Tasks: A Feasibility Study from the North-West Province

**DOI:** 10.3390/s26020705

**Published:** 2026-01-21

**Authors:** Ian Thomson, Mark Kramer

**Affiliations:** 1 Faculty of Engineering, North-West University, Potchefstroom 2520, South Africa; 2 Physical Activity, Sport, and Recreation (PhASRec) Research Focus Area, North-West University, Potchefstroom 2531, South Africa; mark.kramer@nwu.ac.za

**Keywords:** accelerometer, activities of daily living, gyroscope, kinematics, knee function, motion analysis, sensor platform

## Abstract

**Highlights:**

**What are the main findings?**

- A low-cost, instrumented knee brace can accurately measure knee joint angles during various daily activities.
- The device demonstrated good validity when compared with gold-standard motion analysis.

**What are the implications of the main findings?**

- This brace could serve as a cost-effective, accessible tool for monitoring knee function in under-resourced or non-clinical settings.
- Further refinement could make it suitable for clinical use, supporting rehabilitation and movement assessment.

**Abstract:**

Tracking knee joint movement during activities of daily living can have the potential to transform the rehabilitation and functional assessment of patients. The present study evaluated the validity of a low-cost, instrumented knee brace to determine whether it was appropriate for the monitoring and quantification of human knee function during five activity-of-daily-living (ADL) tasks including walking, inclined walking, stepping, sitting, and object manipulation. A sensor platform was designed to acquire sagittal plane knee data from 13 healthy participants across five different tasks and compared to gold-standard motion analysis. The brace showed good-to-excellent validity (RMSE: 4.97–8.65°), with differences in knee joint angles and angular velocities noted during various ADLs, specifically during early and late portions of a given movement. The results for instantaneous knee joint angles and angular velocities were very similar to those of the gold-standard system (mean bias: 0.59–9.52°·s^−1^), which may be applicable to everyday movement tasks, but may preclude analyses at a clinical level. Although the low-cost sensor platform shows promise an effective monitoring tool, it is not ready yet for a clinical application.

## 1. Introduction

Severe ligamentous injuries of the knee, including damage to the anterior cruciate ligament (ACL), frequently necessitate surgical intervention followed by an extended rehabilitation period, typically lasting between 9 and 12 months [1]. During the early phases of recovery, individuals commonly use restrictive knee braces to limit joint motion and protect vulnerable soft tissue structures, thereby supporting effective rehabilitation and gradual tissue adaptation [2]. As rehabilitation progresses, mechanical loading on the knee is progressively increased, accompanied by a corresponding expansion in the functional range of joint motion. Intriguingly, although ACL patients tend to wear a knee brace during the early stages of rehabilitation, little is known about the functional, real-world demands on the knee of these individuals during various activities of daily living (ADL) outside of clinical settings. Knowledge of the functional ability of the ACL-deficient knee is therefore, at least partially, contingent on adherence to the rehabilitation program and careful documentation by the relevant clinician [3,4].

Adherence to prescribed rehabilitation programmes is frequently suboptimal and is influenced by multiple factors, including patients’ perceptions of their symptoms, their evaluation of the perceived effectiveness of the intervention, and the practicality of integrating rehabilitation protocols into daily life [5]. Although real-world knee kinematics can be evaluated using technologies such as video analysis, inertial measurement units (IMU), and motion analysis systems, these methods are typically too restrictive in terms of accuracy, time, and/or money [6]. Despite these limitations, there remains a clear need to assess knee joint function across a range of activities of daily living using low-cost technologies that demonstrate acceptable validity and reliability in both healthy and clinically compromised knees, thereby supporting more informed clinical decision-making by practitioners and rehabilitation specialists [7,8]. From an engineering standpoint, it is important to establish whether a wearable system, such as an instrumented knee brace, can reliably monitor and quantitatively assess knee joint function across a range of activities of daily living. While previous investigations have employed potentiometers, accelerometers, and gyroscopes to capture kinematic data with relatively low measurement error (e.g., root mean square error [RMSE] < 5°), these assessments have typically been limited to a narrow set of movements, most commonly level walking [9]. A need therefore remains for the development of a low-cost brace that can incorporate wearable sensors to capture knee kinematics while completing a diverse range of ADLs, such as (i) ascending and descending slopes, (ii) ascending and descending stairs, (iii) tying a shoe, (iv) walking, and (v) completing a sit-to-stand task.

Moreover, the accessibility to technologies that enable the functional monitoring of individuals is severely restricted in under-resourced clinical settings [10]. Recent evidence has shown that there are less than 10 skilled practitioners per 1 million individuals in low- and middle-income countries [11]. Moreover, in more rural settings, only a few health facilities are available with few practitioners, coupled with a lack of resources. Therefore, for healthcare to be effective, good quality and easily accessible services should be available. Clearly, a gap exists for access to low-cost, yet effective tools for the monitoring of various conditions, of which orthopaedic abnormalities (such as those affecting the knee joint) are a central component.

The primary objectives of the present study were therefore 3-fold: (i) to design a low-cost, instrumented knee brace, (ii) to evaluate sagittal knee joint kinematics during five specific ADLs, and (iii) to establish the validity of the sagittal kinematics from the brace during the specific ADL tasks.

## 2. Materials and Methods

### 2.1. Participants

An a priori sample size of 15 participants was selected based on the kinematic data acquisition requirements of the study [12]. Because participant-specific outcomes (e.g., knee joint range of motion) were directly compared between the instrumented knee brace (reference method) and the Qualisys motion capture system (criterion method), a paired analytical approach was employed to enhance statistical sensitivity [13]. Statistical power was set at 80%, with a corresponding type II error rate of 20%. For kinematic and kinetic analyses, existing recommendations indicate that the collection of approximately 15–20 steps per participant is sufficient to achieve adequate statistical power [14].

For inclusion in the present study, participants had to satisfy the following inclusion criteria: (i) aged 18–45 years, (ii) have no current injuries or history of recent surgery (<6 months) that could influence the ability to perform specific activities of daily living, (iii) must be able to follow all instructions and be in a physical and mental state to complete the informed consent form, and (iv) weigh less than 150 kg (due to limitations in the dimensions of the knee brace). Exclusion criteria included the presence of medical conditions or disorders that could adversely affect gait or the performance of activities of daily living (e.g., osteoarthritis or rickets), as well as pregnancy, due to a potential risk of falls.

The study was approved by the University Ethics Committee and conformed to all principles of the Declaration of Helsinki. The benefits, potential risks, and testing procedures associated with the study were conveyed to all participants both verbally and in writing. All participants read and signed the informed consent form.

### 2.2. Testing Procedures

There were two components to testing, firstly the use and placement of a novel instrumented knee brace, and secondly the use of 3D motion analysis to simultaneously capture detailed kinematics during specific ADLs for validation of the data from the knee brace. The ADLs of interest included:

#### 2.2.1. Tying a Shoe

This required participants to stand in front of a 0.30 m step, place the foot of the instrumented leg on the step and tie a shoelace before returning the foot back to the ground.

#### 2.2.2. Stair Ascent and Descent

Participants were required to ascend a step (0.30 m in height) leading with the instrumented leg pause briefly at the top, and then descend, again leading with the instrumented leg.

#### 2.2.3. Sit-to-Stand

Participants stood facing away from a conventional chair (0.60 m height) and were required to assume a seated position before returning to a standing position.

#### 2.2.4. Slope Ascent and Descent

This task required participants to ascend a slope of 13° in approximately 2–3 strides. Once at the top, participants turned 180° and descended the steps, leading with the instrumented leg until reaching the bottom of the slope.

#### 2.2.5. Level Walking

The simplest task required participants to walk three strides with the instrumented leg.

In all instances, a total of 5 trials were recorded for each participant to capture approximately 15 steps with the instrumented leg for each activity. All trials were ensemble averaged for statistical analysis and comparison.

### 2.3. Knee Brace

**Figure 1:**
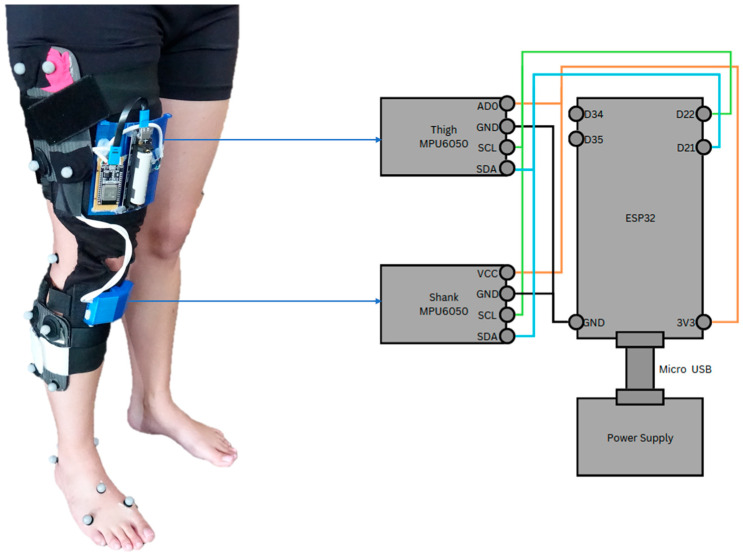
Knee brace sensor platform with motion analysis markers (left). The technical details of the knee brace layout used for the sensor platform are also shown (right).

A knee brace was designed as a platform for the wearable sensors. The knee brace incorporated a total of 2 IMUs (MPU6050, InvenSense, Sunnyvale, CA, USA), one above and one below the knee joint. The brace was attached to the participant with 3 Velcro straps above the knee and 2 Velcro straps below the knee joint. The above-knee IMU, along with the microcontroller (ESP32, Espressif Systems, Shanghai, China) was enclosed in a 3D-printed enclosure and attached to the brace using Velcro above the knee joint (see Figure 1). The below-knee IMU was attached in a 3D-printed enclosure below the knee joint. The accelerometers were used to independently compute the above-knee (thigh) and below-knee (shank) segment angles using Equation (1):

(1) $$Tilt=atan2\;(A_{x}/A_{y})$$

A four-quadrant inverse tangent function (atan2) was used to ensure correct angle estimation across all quadrants. The joint angle was then calculated by using Equation (2):

(2) $$\theta =\theta_{Thigh}-\theta_{Shank}$$

The formulation of the joint angle depended on the quadrant through which the accelerometer rotated. To approximate the anatomical knee joint axis, two accelerometers were mounted on the brace, with one positioned proximal and the other distal to the joint. Both sensors were configured with a full-scale range of ±2 g and a sensitivity of 16,384 LSB/g (see the MPU6050 datasheet for details). It should be noted that inertial measurement units, including accelerometers and gyroscopes, are susceptible to drift over time as a result of temperature variations. To minimise the influence of temperature-related drift, all measurements were conducted in a climate-controlled laboratory. In addition, prior to each trial, participants assumed a brief static standing posture to establish a consistent reference orientation for the sensors. No explicit drift quantification or correction algorithm was applied in this feasibility study. One approach to maintaining a stable temperature is the use of a temperature-controlled environment. This was achieved in the present study by ensuring that all data were collected in a climate-controlled laboratory (temperature: 23 °C; humidity: 55%).

Knee joint angular velocity was measured using the integrated gyroscopes. The gyroscopes were configured with a full-scale range of ±250°·s^−1^ and a sensitivity of 131 LSB·(deg.s^−1^)^−1^ (see the MPU6050 datasheet for further details). Joint angular velocity was subsequently computed using Equation (3):

(3) $$\omega =\omega_{Thigh}-\omega_{Shank}$$

No additional digital filtering was applied to the IMU signals beyond the on-board sensor configuration. A target sampling rate of 20 Hz was selected as a pragmatic, low-cost design choice intended to capture knee kinematics during activities of daily living.

### 2.4. Motion Analysis

Prior to data collection, the capture volume was calibrated using an L-frame (to define the origin of the capture volume) and calibration wand (to digitally orientate the cameras relative to each other and the capture volume). An eight-camera motion capture system was used to acquire kinematic data at a sampling frequency of 200Hz (Oqus 300+, Qualisys, Gotenburg, Sweden). The CAST lower-body marker system was used, which consisted of a total of 34 markers placed on specific anatomical landmarks: anterior superior iliac spine, posterior superior iliac spine, greater trochanter (calibration), thigh cluster, medial and lateral femoral condyles (calibration), shin cluster, medial and lateral malleoli (calibration), base of first ray, head of second metatarsal, base of fifth metatarsal, and calcaneus. Two static calibration trials were recorded which enabled the joint centres and axis of rotation to be determined, such that relevant anatomical coordinate systems could be accurately defined. For the dynamic trials, the calibration markers were removed.

### 2.5. Data Processing

All marker trajectories were processed using Qualisys Track Manager (QTM) software (version 21.1, Qualisys, Gotenberg, Sweden) where missing trajectories were gap-filled using kinematic splines. Processed trials were then filtered using a fourth-order, zero-lag Butterworth filter with a cut-off frequency of 6–15 Hz following a detailed residual analysis and visual inspection of the data [15]. The data were exported to Visual3D (version 2022.02.2, C-Motion, Germantown, MD, USA) where sagittal plane joint angles were calculated for each ADL for the instrumented leg.

To enable efficient data acquisition during the experimental protocol, a dedicated user interface was developed to receive data from the instrumented knee brace. Bluetooth communication was selected to facilitate wireless data transmission between the sensor platform and the user interface, allowing continuous real-time interaction. The microcontroller streamed data to the interface in real time. The user interface was implemented in MATLAB (R2021a Update 3 (9.10.0.1684407), 64-bit; The MathWorks, Natick, MA, USA) and designed as a standalone application capable of managing and visualising the incoming data stream from the sensor platform. Its functionality extends to initiating and terminating data generation, as well as preserving the data for subsequent analysis. The application, as depicted in Figure 1, harnesses Bluetooth to gather data from the sensor platform. It then plots the data instantaneously upon the completion of a designated activity of daily living. The functionality of the application is initiated by the input of an activity name. Once the ‘Start’ button is activated, the indicator light transitions from red to green, indicating that the knee angle data is being displayed in real time. Upon completion of the activity, the ‘Stop’ button is activated, triggering the storage of the raw data into a TXT file. To ensure the accuracy and correctness of the captured data, a ‘Plot’ button was included that enabled a graphical representation of all the collected raw data. The application allows for the input of a new activity name, enabling the repetition of the procedure for varied activities. During laboratory testing, Bluetooth communication remained stable, and no noticeable packet loss or interruptions were observed; however, latency and data loss were not formally quantified and may become more relevant during longer-term or real-world use.

Due to corrupted data for 2 participants, their data could not be included in the final analysis, resulting in a final participant pool of 13 individuals.

### 2.6. Statistics

All data are reported as mean ± standard deviation (SD) unless stated otherwise. Normality of the data was assessed using the Shapiro–Wilk test, with deviations from normality identified at an alpha level of 0.05.

For the first study objective, sensor data were transmitted at a baud rate of 115,200 and stored in XLS format. Data processing was performed in MATLAB (R2021a Update 3 (9.10.0.1684407), 64-bit; The MathWorks, Massachusetts, USA), where outliers were identified and removed, and mean values for each trial were subsequently retained for analysis. For each activity, kinematic signals were ensemble averaged and analysed as a function of time, with temporal normalisation applied to enable comparisons across participants and tasks.

For the second study objective, triaxial measurements obtained from the instrumented knee brace were compared with data acquired from the three-dimensional motion capture system and force plates. Differences in angular kinematics between the knee brace and the Qualisys system were evaluated using a paired *t*-test implemented within the statistical parametric mapping framework (SPM{t}; Python, SPM1d, v. 0.4.18). The SPM{t} approach is grounded in random field theory, which characterises the probabilistic behaviour of smooth, time-varying signals and was used to define a critical significance threshold (α = 0.05). Angular data were considered significantly different at time points where the SPM{t} trajectory exceeded this threshold. In addition, validity was assessed using (i) Bland–Altman analysis to determine the level of agreement between peak kinematic values obtained from the knee brace and the Qualisys motion capture system (criterion method), with agreement within 5% considered acceptable, and (ii) the RMSE for the full curve to evaluate the mean discrepancy between the reference and criterion measures, where an RMSE discrepancy of <5° was considered excellent, and 5–10° was considered good [16,17]. To facilitate the qualitative interpretation of angular velocity measures (ω), we also incorporated the RMSE-observed standard deviation ratio (RSR) which is calculated as the ratio of the RMSE and standard deviation of measured data [18]. The qualitative thresholds for the RSR are as follows: very good: ≤0.50; good: 0.51–0.60; satisfactory: 0.61–0.70; unsatisfactory: >0.70. Finally, to facilitate the practical utility of the differences between devices, we have also added the minimal detectable change (MDC) and standard error of measurement (SEM) as these present thresholds beyond which differences likely reflect true disagreement between devices rather than measurement noise. All statistical tests were completed using RStudio (2023.05.0, Build 496, Posit Software, Boston, MA, USA, PBC).

## 3. Results

### 3.1. Walking Trials

**Figure 2:**
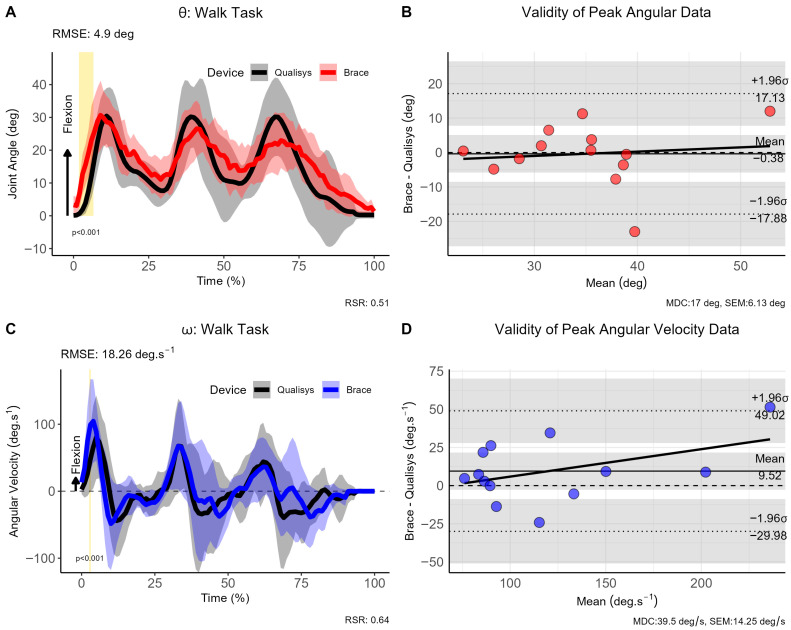
Comparison of brace- and motion capture joint angles and angular velocities during walking. (**A**) Instantaneous joint angles, (**B**) validity of peak joint anlges, (**C**) instantaneous joint velocities and (**D**) validity of angular velocity data for the walking trials. Note: θ: joint angle (in degrees [deg]); ω: angular velocity (in degrees per second [deg.s^−1^]); σ: standard deviation; CI: 95% confidence interval; RMSE: root mean square error; vertical light-golden shaded area indicates regions of statistically significant differences; MDC = minimal detectable change; SEM = standard error of measurement.

The knee joint range of motion (ROM; θ), angular velocity (ω), and Bland–Altman analyses for the walking trials are shown in Figure 2. Although there is considerable overlap between the reference and criterion methods in both θ and ω data, the SPM analysis shows that there were significant differences over the first 3–7% of the gait cycle for θ (*p* < 0.001), and 2–4% for ω (*p* < 0.001). The RMSE results show that the discrepancy between methods is excellent on the basis that the mean error is <5° and the mean error in ω is ~18°·s^−1^. With regards to the peak θ and ω kinematic values, the Bland–Altman analysis showed that there was moderate bias in θ (−0.38°, 95% CI [−17.88, 17.13]) and ω (9.52°·s^−1^, 95% CI [−49.02, 29.98]).

### 3.2. Tying Shoelace Trials

**Figure 3:**
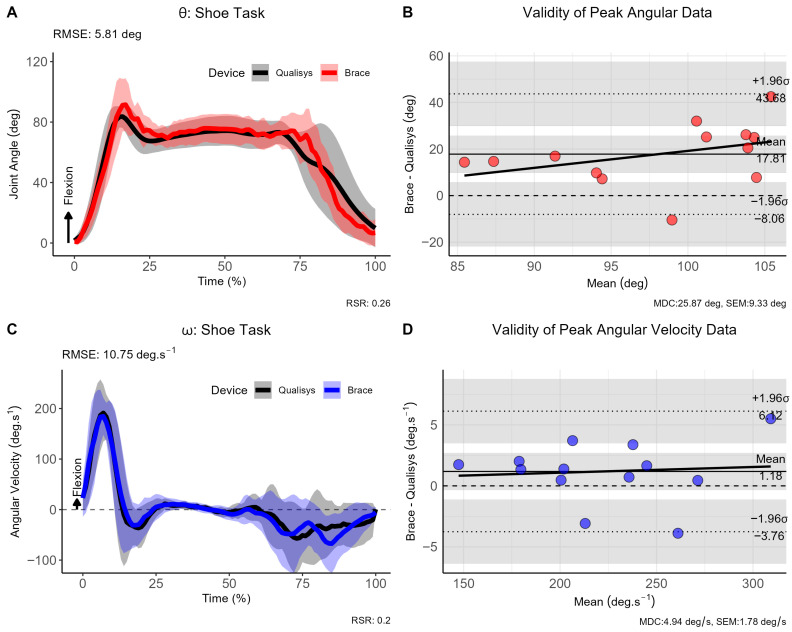
Comparison of brace- and motion capture joint angles and angular velocities during the shoelace tying task. (**A**) Instantaneous joint angles, (**B**) validity of peak joint anlges, (**C**) instantaneous joint velocities and (**D**) validity of angular velocity data across the shoelace tying task. Note: θ: joint angle (in degrees [deg]); ω: angular velocity (in degrees per second [deg.s^−1^]); σ: standard deviation; CI: 95% confidence interval; RMSE: root mean square error; vertical light-golden shaded area indicates regions of statistically significant differences; MDC = minimal detectable change; SEM = standard error of measurement.

Differences between reference and criterion methods for θ and ω were negligible as evaluated by SPM, showing a lack of statistically significant clusters (see Figure 3). The RMSE also showed good consistency throughout the curves with a mean difference of 5.81° for θ and 10.75°·s^−1^ for ω. For the shoelace tying task, bias in peak θ was moderate (17.81°, 95% CI [−43.68, 8.06]), whereas the bias for ω was acceptable (1.18°·s^−1^, 95% CI [−3.76, 6.12]).

### 3.3. Sit-to-Stand Trials

**Figure 4:**
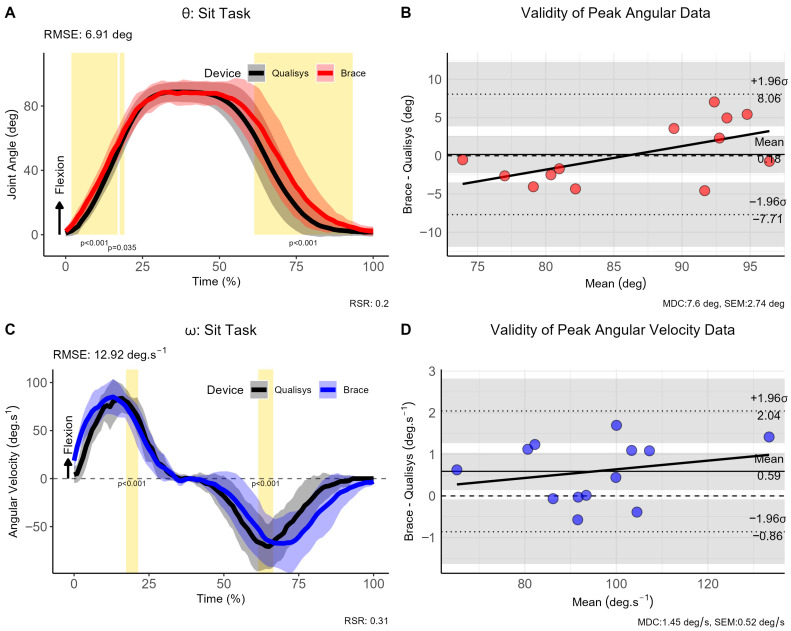
Comparison of brace- and motion capture joint angles and angular velocities during ‘Sit-to-stand’ task. (**A**) Instantaneous joint angles, (**B**) validity of peak joint anlges, (**C**) instantaneous joint velocities and (**D**) validity of angular velocity data for the ‘sit-to-stand’ trials. Note: θ: joint angle (in degrees [deg]); ω: angular velocity (in degrees per second [deg.s^−1^]); σ: standard deviation; CI: 95% confidence interval; RMSE: root mean square error; vertical light-golden shaded area indicates regions of statistically significant differences; MDC = minimal detectable change; SEM = standard error of measurement.

The sit-to-stand task exhibited significant differences during the initial and terminal phases of the movement (see Figure 4). The RMSE showed good consistency throughout the curves with a mean difference of 6.91° for θ and 12.92°·s^−1^ for ω. Bias in peak θ (0.18°, 95% CI [−7.71, 8.06]), and ω was very low (0.59°·s^−1^, 95% CI [−0.86, 2.04]), although the SPM analysis revealed statistically significant differences at the beginning and end of the sitting movement for θ (*p* < 0.001), and the mid-portions of ω (*p* < 0.001).

### 3.4. Stair Climb Trials

**Figure 5:**
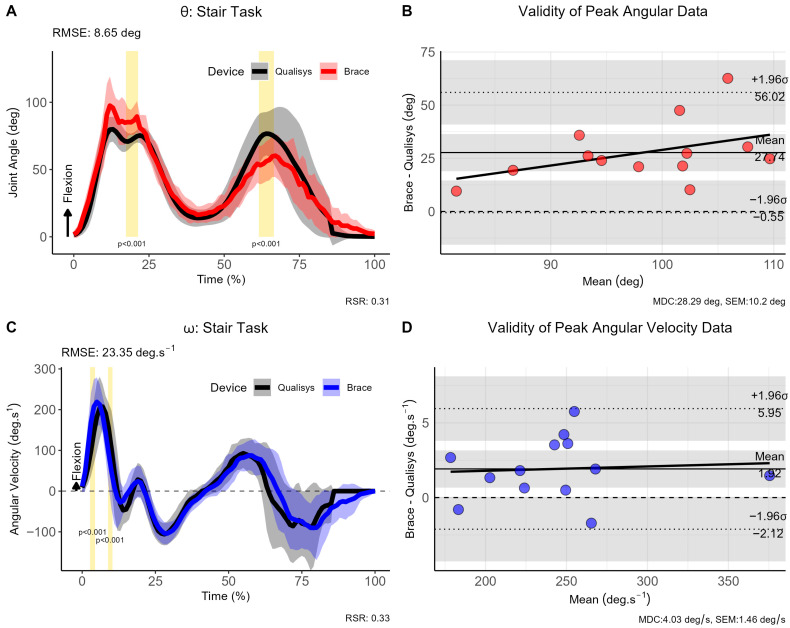
Comparison of brace- and motion capture joint angles and angular velocities during stair climb task. (**A**) Instantaneous joint angles, (**B**) validity of peak joint anlges, (**C**) instantaneous joint velocities and (**D**) validity of angular velocity data for the stair climb trials. Note: θ: joint angle (in degrees [deg]); ω: angular velocity (in degrees per second [deg.s^−1^]); σ: standard deviation; CI: 95% confidence interval; RMSE: root mean square error; vertical light-golden shaded area indicates regions of statistically significant differences; MDC = minimal detectable change; SEM = standard error of measurement.

Participants were required to ascend and descend a step of 0.30 m whereby the movement was initiated with the braced leg. The RMSE for the joint angle was 8.65°, indicating good consistency between measurement approaches; however, the mean bias for the peak joint angle was 27.74° (95% CI [−0.55, 56.02]) indicating a substantial shift. Similarly, the RMSE for ω was 23.35°·s^−1^ with a smaller systematic bias of just 1.92°·s^−1^ (95% CI [−2.12, 5.95]) (see Figure 5). The SPM analysis revealed statistically significant differences at the beginning and end of the stair climb movement for θ (*p* < 0.001), and the beginning portion of ω (*p* < 0.001)

### 3.5. Slope Trials

**Figure 6:**
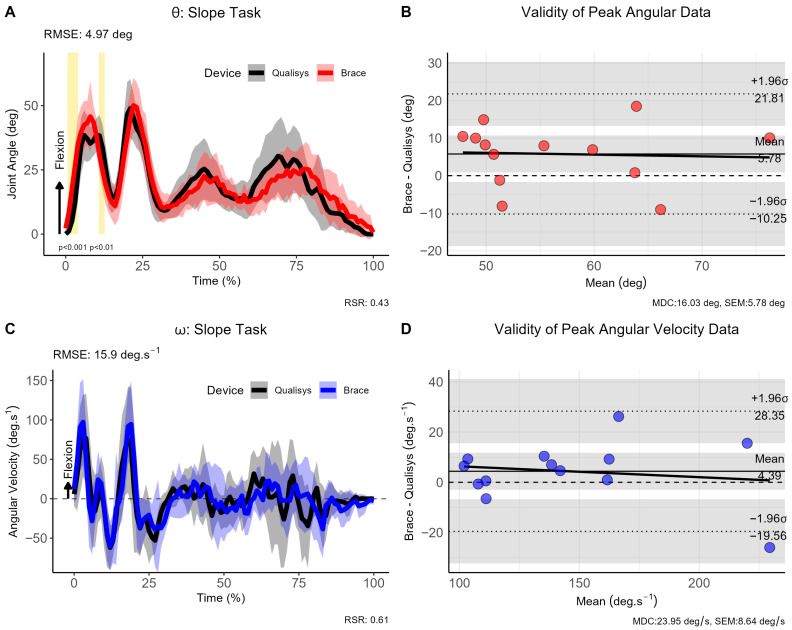
Comparison of brace- and motion capture joint angles and angular velocities during sloped walking trials. (**A**) Instantaneous joint angles, (**B**) validity of peak joint anlges, (**C**) instantaneous joint velocities and (**D**) validity of angular velocity data for the sloped walking trials. Note: θ: joint angle (in degrees [deg]); ω: angular velocity (in degrees per second [deg.s^−1^]); σ: standard deviation; CI: 95% confidence interval; RMSE: root mean square error; vertical light-golden shaded area indicates regions of statistically significant differences; MDC = minimal detectable change; SEM = standard error of measurement.

For the slope trials, participants were required to ascend and descend a slope of 2 m angled at 13°, whereby the movement was again initiated with the braced leg. The RMSE for the joint angle was 4.97° (95% CI [−22.04, 13.04]), indicating excellent consistency between measurement approaches, whereas a mean difference of 15.90°·s^−1^ was observed for ω (95% CI [−27.66, 19.71]) (see Figure 6). The SPM analysis revealed statistically significant differences only for the first step of the slope task for θ (*p* < 0.001), but not for ω.

A summary of the main task-specific RMSE and RSR results for both the joint angle (θ) and angular velocity (ω) data are highlighted in Table 1 for ease of reference.

## 4. Discussion

The primary findings of the present study showed that it was possible to design a valid yet low-cost instrumented knee brace to evaluate sagittal plane knee joint kinematics during real-life ADLs. Access to instrumented knee braces within the African context is currently not feasible on the basis that such devices do not yet exist, especially at a commercial level. The present study is the first to show that, at least within an African context, it is indeed plausible to develop a low-cost knee brace (~R1000 or ~$55) capable of quantifying knee kinematics across a diverse range of environmental obstacles such as slopes, stairs, and chairs.

Previous studies have designed and tested the development of a knee brace, however these have focused exclusively on gait [19,20] or mathematical algorithms for data extraction. The kinematic data provided in the present study were on par with those of previous research [21,22], and showed that there was considerable variability in knee function across different tasks and environmental obstacles (e.g., slopes, stairs, chairs). Knowing the functional demands on the knee, as well as the “true” range of a knee during such varied tasks would be useful to both clinicians and practitioners during rehabilitation and/or conditioning to more objectively target the ability of the knee and the surrounding soft tissue structures. Although the brace showed good-to-excellent validity (see Table 1), differences in knee joint θ and ω were noted during various ADLs, indicating that the performance of the device depended on the complexity of the movement. These phase-dependent discrepancies are likely attributable, in part, to the use of a simplified accelerometer-based tilt estimation without sensor fusion, drift compensation, or three-dimensional orientation modelling. The device performed exceptionally well during continuous rhythmic tasks such as level walking (RMSE: 4.90°, excellent) and slope walking (RMSE: 4.97°, excellent). Moderate performances were observed for shoelace tying (RMSE: 5.81°, good) and the sit-to-stand (RMSE: 6.91°, good) movements which are more controlled, quasi-static tasks. Stair climbing was the poorest performing task (RMSE: 8.65°, good) as it exhibited the highest RMSE and a peak bias of 27.74°. Collectively, these results reveal that the device can track the general movement pattern of various tasks well but struggles to quantify precise magnitudes of tasks requiring deeper knee flexion angles (e.g., shoelace tying and stairs).

Three technical factors likely contributed to the discrepancies observed between the knee brace (reference method) and the motion analysis system (criterion method). Firstly, sensor limitations in wearable devices, such as sensor drift, accuracy, precision, and sampling rate, may affect the reliability of the data [23]. The placement and attachment of these wearable devices can also influence the accuracy of the motion data on the basis that misalignment or loosening of the sensors can cause notable discrepancies in the magnitude of joint angles which may have implications for evaluating the functional ability of the knee during specific activities [24]. Environmental factors also play a role in this divergence whereby lighting conditions and background noise can affect camera-based systems, while wearables may be influenced by electromagnetic interference and temperature changes. Lastly, temporal synchronisation issues might also lead to inconsistencies [25]. The different sampling rates or lack of perfect synchronisation between the wearable and camera-based system can result in inconsistent measurements, further widening the gap between the two data sets. Although all efforts were made to maximise the accuracy of data collection process, the results indicate that some refinement of the brace (e.g., algorithms, Bluetooth unit selection) would be necessary in order to enhance to overall utility of the unit. Sensor drift and temperature-related effects were not explicitly quantified in the present study and should be addressed through formal calibration and correction procedures in future work.

The magnitude of the mean bias and the width of the limits of agreement (LoA) in tasks such as stair ascent/descent suggests that the system is not yet suitable for clinical applications where precise diagnostic measurements are required. However, the good-to-excellent RMSE values indicate that the device is valid for functional monitoring during everyday tasks. As such, in under-resourced or rural settings, this tool could provide valuable feedback on adherence to rehabilitation programs or general movement patterns, filling a critical gap where no objective data currently exists. To bridge the disparity between the use as a functional monitoring tool and a clinical-grade device, several targeted engineering refinements are required. In particular, the implementation of sensor fusion and advanced filtering approaches, such as Kalman-based methods, may improve orientation estimation and reduce noise during dynamic movement. Regular calibration procedures would further assist in mitigating sensor drift and temperature-related effects over time. While these refinements are expected to improve accuracy, they must be carefully balanced against cost considerations to maintain the affordability and accessibility of the device, particularly in under-resourced clinical settings.

Key limitations within the current study must also be mentioned. We were unable to complete a reliability analysis due to participant scheduling conflicts, thereby rendering the final sample size for retesting too small to be meaningful. The brace was tested on a single leg to evaluate the feasibility and accessibility of the device as a ‘proof of concept’. Future iterations should include a brace on both limbs to determine whether between-limb differences (i.e., asymmetries) can be detected within healthy and clinically compromised populations. The effects of sensor fusion and more advanced filtering algorithms should be considered and explored in greater detail. The reliance on Bluetooth technology for the brace versus the high-frequency capture of the camera system likely introduced latency or sampling rate mismatches. This lack of perfect synchronization helps explain the statistical differences observed in the SPM analysis, particularly at the initiation and termination of movements such as the sit-to-stand and stair climbing tasks, where timing offsets result in significant divergence between the two data sets. Finally, the present study was conducted exclusively on healthy participants. While this approach was appropriate for an initial feasibility and technical validation study, the findings cannot be directly generalised to individuals with knee pathology, such as anterior cruciate ligament (ACL) injury or other orthopaedic impairments. Altered movement strategies, joint instability, and reduced ranges of motion in clinical populations may influence both sensor alignment and measurement accuracy.

## 5. Conclusions

This study successfully developed and validated a low-cost, instrumented knee brace capable of quantifying sagittal plane kinematics during a diverse range of ADLs. Our results confirmed that a device constructed for approximately R1000 can achieve good-to-excellent validity (RMSE < 9°) compared to a gold-standard optical motion capture system. While the brace demonstrated high precision during continuous, rhythmic tasks like walking and sloped walking, its accuracy diminished during tasks that involved greater knee flexion and rapid transitions, such as stair climbing and sit-to-stand movements.

The research highlighted critical technical barriers that would hinder clinical adoption, specifically mechanical slippage during leg extension and the inherent latency of Bluetooth-based synchronization. These factors highlight the current role of the device as a robust tool for functional movement monitoring in non-clinical or under-resourced settings, rather than a diagnostic tool requiring absolute angular precision.

To bridge this gap, future research must move beyond basic sensor integration. Refinement of the physical mounting interface is required to mitigate slippage, while the implementation of advanced sensor fusion (e.g., Kalman filtering) and machine learning algorithms could significantly reduce data noise and drift. Overall, this research establishes a foundational framework for accessible, wearable biomechanical analysis, offering a scalable solution for long-term rehabilitation monitoring and movement assessment in diverse global contexts.

## Figures and Tables

**Table 1:** Summary and Interpretation for task-specific RMSE and RSR.

Task	Joint θ RMSE (RSR)	Qualitative Interpretation θ	Joint ω RMSE (RSR)	Qualitative Interpretation ω
Walk	4.90° (0.51)	Excellent	18.26°·s^−1^ (0.64)	Good
Shoelace	5.81° (0.26)	Good	10.75°·s^−1^ (0.20)	Very Good
Sit	6.91° (0.20)	Good	12.92°·s^−1^ (0.31)	Very Good
Stair	8.65° (0.31)	Good	23.85°·s^−1^ (0.33)	Very Good
Slope	4.97° (0.43)	Excellent	15.90°·s^−1^ (0.61)	Good

Note: RMSE = root mean square error; RSR = RMSE-observed standard deviation ratio.

## Data Availability

The data for this article are freely available at the following link: https://doi.org/10.7910/DVN/GTTPDS.

## Abbreviations

The following abbreviations are used in this manuscript.

ADL	Activities of Daily Living
ACL	Anterior Cruciate Ligament
IMU	Inertial Measurement Unit
RMSE	Root Mean Square Error
SPM	Statistical Parametric Mapping
QTM	Qualisys Track Manager
